# The Parahippocampal Cortex Mediates Contextual Associative Memory: Evidence from an fMRI Study

**DOI:** 10.1155/2016/9860604

**Published:** 2016-05-09

**Authors:** Mi Li, Shengfu Lu, Ning Zhong

**Affiliations:** ^1^International WIC Institute, Beijing University of Technology, Beijing 100124, China; ^2^Beijing International Collaboration Base on Brain Informatics and Wisdom Services, Beijing 100124, China; ^3^Beijing Key Laboratory of MRI and Brain Informatics, Beijing 100053, China; ^4^Department of Life Science and Informatics, Maebashi Institute of Technology, Maebashi 371-0816, Japan

## Abstract

The parahippocampal cortex (PHC) plays a key role in episodic memory, spatial processing, and the encoding of novel stimuli. Recent studies proposed that the PHC is largely involved in contextual associative processing. Consequently, the function of this region has been a hot debate in cognitive neuroscience. To test this assumption, we used two types of experimental materials to form the contextual associative memory: visual objects in reality and meaningless visual shapes. New associations were modeled from either the contextual objects or the contextual shapes. Both contextual objects and shapes activated the bilateral PHC more than the noncontextual ones. The contextual objects with semantics significantly activated the left PHC areas, whereas the meaningless contextual shapes significantly elicited the right PHC. The results clearly demonstrate that the PHC influences the processing of contextual information and provides experimental evidence for an understanding of the different functions of bilateral PHC in contextual associative memory.

## 1. Introduction

Visual objects in the environment tend to exhibit relationships (e.g., a football with a football field, a cow with a farmer, and a supermarket cart with a supermarket); that is, relationship is a basic property in nature. Therefore, studying brain function in associative information processing is important for understanding the cognitive mechanisms of the human brain. Previous studies demonstrated that the hippocampus and the parahippocampal cortex (PHC) play different roles in cognitive processing. The hippocampus is considered to be related to associative learning and memory [1–7], whereas the PHC is more involved in spatial processing [8–16], episodic memory [17–23], and encoding of novel stimuli [24–28]. Recently, Bar et al. performed a series of functional magnetic resonance imaging (fMRI) experiments and proposed that the PHC was strongly involved in contextual associations [29–32]. They compared objects comprising strong contextual associations with those consisting of weak associations and found significant activation in the PHC [29, 30]. To support their theory, they compared the activation elicited by famous faces with that elicited by unfamiliar faces and found significant activation in the PHC [32], which indicates that the participants unconsciously associated the pictures with contextual information about these famous people. This observation is due to the familiarity of the participants with the background of these famous people. In contrast, in an experiment involving unfamiliar face perception, the participants formed no mental contextual information. Epstein and Ward repeated the experiment using famous faces and found no significant activation in the PHC; therefore, they doubt the reliability of the visual contextual effects in the PHC [33]. In the study by Bar and Aminoff [29], all strong contextual tasks were associated with specific episodic memory (e.g., associating a sun lounge with the beach, a microscope with a laboratory, and a gas stove with a kitchen), whereas weak contextual objects were not associated with any specific context (e.g., an apple, a Rubik's cube, and camera). Contextual processing in their study (strong contextual objects versus weak contextual objects) was highly similar to episodic memory and spatial information processing; the results were also affected by differences in living environment, experiences, and prior knowledge among participants. Thus, the role of the PHC in visual contextual associative processing should be investigated.

We also investigated the different functions of bilateral PHC in contextual associations using two types of experimental materials: visual objects with semantic meaning and meaningless visual shapes. The bilateral hemispheres of the human brain perform different functions: the left hemisphere is more involved in cognitive processing, such as language comprehension and logical reasoning, whereas the right hemisphere is more involved in shape recognition and visual spatial processing. Previous studies on associative memory mainly focused on the associations between objects and spatial locations and found that the right PHC is more significantly activated than the left PHC in the associative memory of object location [31, 34, 35]. Another study on verbal recognition tasks showed that the left and right PHC had different roles [36]. Thus, we expected the role of bilateral PHC to be different, that is, the left PHC to be activated more by contextual objects and the right PHC by contextual shapes.

In the current study, there are two main aims: the first one is to investigate the role of the PHC in visual contextual associative processing and the second is to address the different functions of bilateral PHC in contextual associations of the visual objects and visual shapes.

## 2. Methods

### 2.1. Subjects

A total of 16 right-handed healthy subjects, 7 females and 9 males, with a mean age of 22.5 years (SD 1.8), a mean educational level of 16.5 years (SD 0.6), and normal or corrected-to-normal vision, were enrolled in the study. None reported any history of neurologic or psychiatric diseases. Written informed consent was obtained from each participant prior to the scanning and training sessions. All procedures were approved by the Ethical Committee of the Xuanwu Hospital of Capital Medical University. None of the subjects had previously participated in similar experiments.

### 2.2. Stimuli

We selected objects with very weak association with any context and trained the subjects to form a direct relationship between two paired objects. This method minimizes the effects of possible differences among individual environments and experiences. Prior to fMRI scanning, all subjects were required to participate repeatedly in the training and testing phases of associative memory until they formed a solid contextual associative memory. This method eliminates the effects of individual prior knowledge. Additionally, to completely disregard the contextual episodic memory that may be present in weak contextual objects, we created meaningless visual shapes that are considered novel to all participants [31]. These meaningless stimuli efficiently control the formation of episodic memory. During the training period, two objects (shapes) were shown at random locations on the screen to avoid associative memory between objects and spatial locations. All participants were exposed equally to all training stimuli. After two weeks of training, all participants were familiar with these meaningless shapes, thereby eliminating novel effects. Therefore, the use of both visual contextual objects and shapes would activate the PHC more than the noncontextual stimuli. We verified that the activation of the PHC was attributed to contextual associations rather than episodic memory, spatial processing, and encoding of novel stimuli.

The stimuli (objects and shapes) consisted of black-white images on a black background. Object images with weak contextual association were taken from the normative data to collect pictures using the method by Snodgrass and Vanderwart [37]. Shape images consisted of line drawings that were created and selected to trigger no explicit semantic meaning. The stimuli spanned a 4° visual angle, presented within a 12° black square span. The black square was divided into nine sections, where each stimulus could be presented.

**Figure 1:**
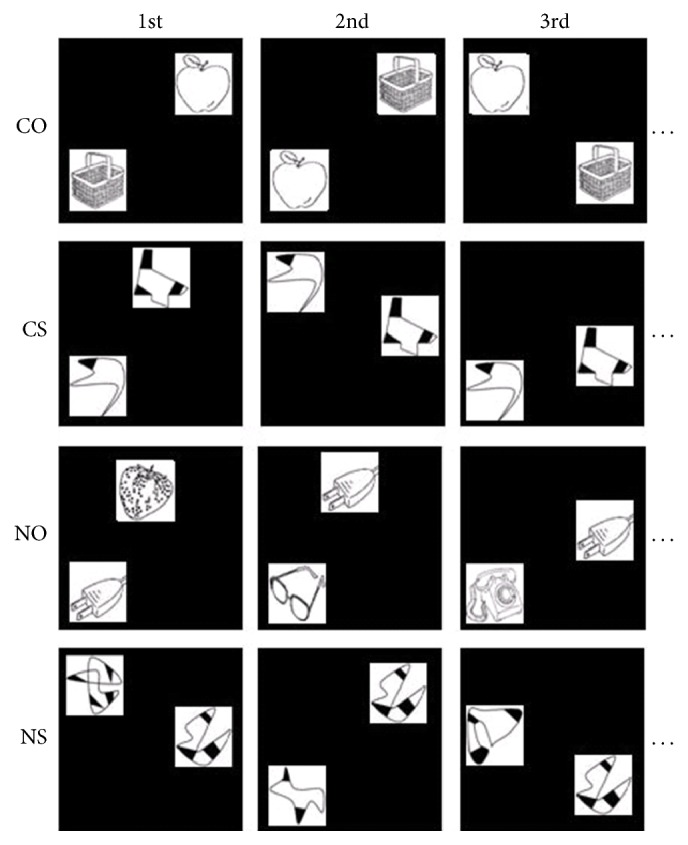
Examples of displays in the training trials from different sessions (first, second, and third presentation, and so on). COs: contextual objects; CSs: contextual shapes; NOs: noncontextual objects; NSs: noncontextual shapes.

The experiment included four conditions: contextual object (CO), noncontextual object (NO), contextual shape (CS), and noncontextual shape (NS). Each condition included 80 stimuli. For each contextual associative condition (objects or shapes), the stimuli in each pair were grouped together; thus, the 80 stimuli of CO or CS were divided into 40 groups. For the noncontextual associative condition, the stimuli were grouped randomly. During the training period, the groups of two objects or shapes under the contextual associative conditions were always presented together, whereas the groups of two similar, noncontextual associative stimuli were presented only once. Under all conditions, the stimuli were presented at random locations on the screen. Examples of these stimuli are shown in Figure 1.

### 2.3. Training

The training aims to form contextual associative memory between two objects or two shapes. The training period consisted of a study phase and a testing phase. To ensure that the associations were well-established, the mean training period continued for two weeks, with an hour-long session each day. The training period was determined by the performance of the participants; therefore, the training schedule varied for each participant.

During the study phase, the participants passively viewed the stimuli, which appeared as either pairs of objects or pairs of shapes. Under the contextual associative conditions, two contextual stimuli (objects or shapes) were always presented together, whereas, under the noncontextual associative conditions, two noncontextual stimuli (objects or shapes) were presented only once. Under all conditions, the stimuli were presented in pairs to ensure associative and nonassociative learning during the same session. To avoid association of the stimuli with a fixed location, the screen was divided into 9 sections wherein every paired stimulus was presented at two random locations. The stimuli presented during the training phase were from 4 categories: COs, CSs, NOs, and NSs.

The study phase consisted of three stimulus repetitions. Each stimulus was presented once and repeated three times. The duration of stimuli presentation was determined by the participants. When participants became more proficient with the stimuli (indicated by a test score of at least 80), they received only one repetition. The viewing during the study phase was self-paced, and the stimuli presentation was controlled by the participant. The participants proceeded to the testing phase after the study phase of each training session.

The testing phase consisted of two types of quizzes: categorical quiz and multiple-choice quiz. In the categorical quiz, one object or shape was presented, and the participant had to press a button to report whether the object or shape has a contextual or noncontextual association. The participants were given feedback on response accuracy after each decision. Participants who became more proficient with the stimuli (test score ≥ 80) were required to take the multiple-choice test. In this test, one stimulus (object or shape) was presented on the upper portion of the screen, and options from A to D (objects or shapes) were presented on the lower portion. The participants were instructed to press a corresponding button to respond. For contextual associative stimuli, the participants had to select the associated stimulus; otherwise, the participants pressed the space bar and moved on to the subsequent item. To vary the stimuli selected in each quiz, the tasks were designed in two ways: first, the stimulus target was changed, and then, options from A to D were changed. This process eliminated the effects of familiarity on testing performance. After each correct answer, the participants proceeded to the next item; otherwise, they were provided with the correct answer to improve their memory performance. In the categorical and multiple-choice tests, we observed two conditions: (a) for contextual stimuli (COs and CSs), not all stimuli represented both the categorical and multiple-choice test. For example, for *X*_*i*_ and *Y*_*i*_ to be associative, denoted as *X*_*i*_ − *Y*_*i*_, *i* = 1, 2,…, *m*, the following m associative groups would be required: *X*_1_ − *Y*_1_, *X*_2_ − *Y*_2_, *X*_3_ − *Y*_3_, …, *X*_*m*_ − *Y*_*m*_. In the test, the subjects were divided into two groups: group A and group B. For group A, *X*_*i*_ was used in the categorical test, and *Y*_*i*_ was used in the multiple-choice test. For group B, *Y*_*i*_ was used in the categorical test, and *X*_*i*_ was used in the multiple-choice test. (b) For noncontextual stimuli (NOs and NSs), all of the stimuli were used in the categorical test and not the multiple-choice test. Participants who performed with >95% accuracy in all test sections were assumed to have learned the associations effectively and were allowed to proceed to the fMRI experiment.

### 2.4. fMRI Experimental Procedure

**Figure 2:**
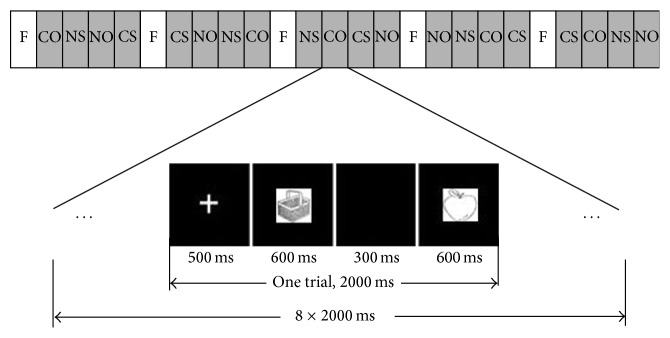
Paradigm of stimulus presentation in the fMRI experiment. F: fixation block; COs: contextual objects; CSs: contextual shapes; NOs: noncontextual objects; NSs: noncontextual shapes. Each block was 16 s in length, with each block consisting of 8 trials (presented for 2000 ms).

Four conditions were established in the fMRI experiment: CO, CS, NO, and NS. A total of 160 trials were conducted (40 trials per condition). A trial consisted of a fixation dot (+) shown for 500 ms, followed by one of four targets randomly chosen (objects or shapes) shown for 600 ms. The target was immediately followed by a mask (shown for 300 ms), which was replaced with a black screen and finally a test stimulus (shown for 600 ms). The total length of each trial was 2000 ms. Under each condition, 40 trials were equally distributed into 5 blocks, with each block consisting of 8 trials (16 s). A total of 20 blocks under the 4 conditions were set, and one fixation block (16 s) was interleaved after every 4 stimulus blocks (Figure 2). In the presentation of the target stimulus or test stimulus, the subjects were instructed to classify the stimulus as contextual or noncontextual and to press the corresponding button. If a target stimulus was contextual, they would press the left button; otherwise, they pressed the right button. If a test stimulus was associated with the target stimulus, the subject would press the left button; otherwise, they pressed the right button.

### 2.5. Imaging Parameters

Scanning was performed on a 3.0 T Siemens system by using a standard whole-head coil. Functional data were acquired using a gradient echo planar pulse sequence (TR = 2000 ms, TE = 31 ms, flip angle = 90°, voxel size = 3.75 mm × 3.75 mm × 4 mm, 30 slices, slice thickness = 4 mm, interslice interval = 0.8 mm, matrix = 64 × 64, and FOV = 240 mm × 240 mm). T1-weighted anatomical images were collected on the same plane as the functional image using a spin echo sequence with the following parameters: TR = 130 ms, TE = 2.89 ms, flip angle = 70°, voxel size = 0.8 mm × 0.8 mm × 4 mm, 30 slices, slice thickness = 4 mm, interslice interval = 0.8 mm, matrix = 320 × 320, and FOV = 240 mm × 240 mm. The stimuli were presented, and the data were synchronized using E-Prime 2.0 (Psychology Software Tools, Pittsburgh, USA). The scanner was synchronized with every trial presentation in each run.

### 2.6. Data Analysis

The fMRI data were analyzed using the SPM 8 software (Welcome Department of Cognitive Neurology, London, UK, http://www.fil.ion.ucl.ac.uk/). Prior to each run, the first two discarded volumes were acquired to stabilize magnetization. Standard preprocessing of functional images was performed, including slice timing correction, rigid-body motion correction and unwarping, spatial normalization to the standard MNI template (resampled at 2 mm × 2 mm × 2 mm), and spatial smoothing (using an 8 mm full-width half-maximum isotropic Gaussian kernel). The data underwent high-pass filtering to consider low-frequency drift, with a cut-off value of 160. In the first level of statistical analyses (single subject), the least squares parameter estimates of the height of the best fitting synthetic HRF for each condition were used in pairwise contrasts and were stored as a separate image for each subject. In the second level of statistical analyses (group analysis), a random-effects model was used. The images were then tested against the null hypothesis that there is no difference between conditions using one-sided *t*-tests. To test the involvement of PHC in the associative processing, we performed direct comparisons of CO versus NO and CS versus NS. To test the involvement of PHC between objects with semantics and shapes without semantics, we compared CO versus CS and NO versus NS. Moreover, to determine the PHC involvement in contextual associative processing, a main-effect analysis [(CO + CS) versus (NO + NS)] was performed. To obtain an accurate result, a region was considered significant if 10 or more contiguous voxels (80 mm^3^) were present and if the alpha threshold (*P* < 0.05, corrected) was exceeded.

ROI analysis was based on the Harvard-Oxford probabilistic map. We defined two functional ROIs of the peak voxels in left and right PHC using the MarsBar software (http://marsbar.sourceforge.net/) and extracted the mean time course across activated voxels in each ROI for each participant. The percent signal change was calculated individually for each subject using that subject's fixation activation as baseline and then averaging across subjects. In the region-of-interest (ROI) analysis, we defined the extent of the PHC ROI using the Harvard-Oxford atlas (http://fsl.fmrib.ox.ac.uk/fsl/fslwiki/Atlases) and MRIcron (http://www.mccauslandcenter.sc.edu/mricro/mricron). Voxels were included if the atlas labeled them as “parahippocampal cortex, anterior division” or “parahippocampal cortex, posterior division” with a probability of >25% [38–40]. To further analyze the results, we conducted a 3-way ANOVA (association, semantics, and hemisphere; 2 × 2 × 2) with repeated measures analysis on the BOLD signal changes of the left and right PHC using ROI analysis. We also did the two-by-two comparisons in the bilateral PHC, respectively, and the Bonferroni method was applied to conduct multiple comparison corrections.

## 3. Results

### 3.1. Behavioral Results

The participants were trained for an average period of two weeks. Those who consistently performed above 95% in the training tests under all conditions were allowed to proceed with the fMRI experiment. The fMRI experiment indicated no significant difference in accuracy among the four conditions: CO, CS, NO, and NS. Two-way analysis of variance (ANOVA) with repeated measures showed that the main effect of semantics was a little lower than the significant level, *F*(1, 15) = 2.980, *P* = 0.089; the main effect of the contextual factor was not significant, *F*(1, 15) = 0.025, *P* = 0.421; and the interaction between the two factors was also not significant, *F*(1, 15) = 0.616, *P* = 0.436. As shown in Table 1, the accuracy rates under both object conditions (CO and NO) were higher than those under the shape conditions (CS and NS). The higher accuracy rates might be related to the objects with the semantic labels, which helped the participants to form visual memory easily and to improve the accuracy rate of perceptual judgments.

### 3.2. fMRI Results

**Figure 3:**
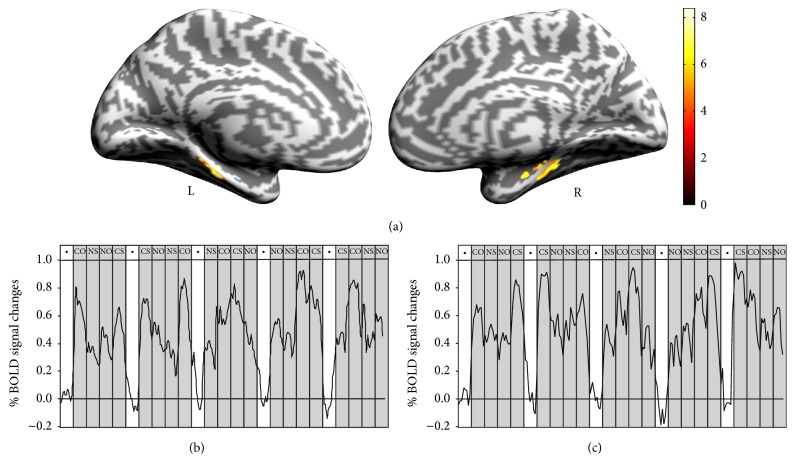
Main-effect analysis. (a) Bilateral PHC activation maps for the main effect of contextual associative memory [(CO + CS) versus (NO + NS)]. (b) The time course of the percent changes in a peak voxel in the left PHC (MNI: −28/−30/−24, *z* = 5.56) over the period of the scan. (c) The time course of the percent changes in a peak voxel in the right PHC (MNI: 32/−32/−18, *z* = 5.72) over the period of the scan. The percent signal change was calculated individually for each subject using that subject's fixation activation as baseline and then averaging across subjects (black dot indicates fixation epochs). COs: contextual objects; NOs: noncontextual objects; CSs: contextual shapes; NSs: noncontextual shapes; L: left hemisphere; R: right hemisphere.

**Figure 4:**
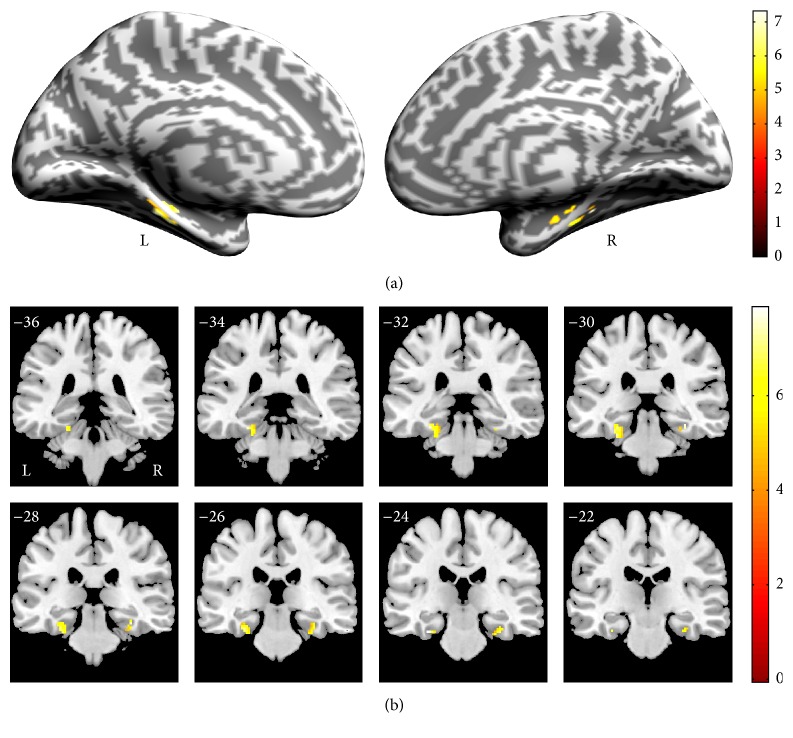
Random-effects statistical activation maps within the PHC for contextual objects versus noncontextual objects. (a) Cerebral cortex inflation of the left and right hemisphere. (b) Coronal sections. The numbers of the coronal sections refer to the coordinates of coronal orientation in accordance with the Montreal Neurological Institute (MNI) space; L: left hemisphere; R: right hemisphere.

**Figure 5:**
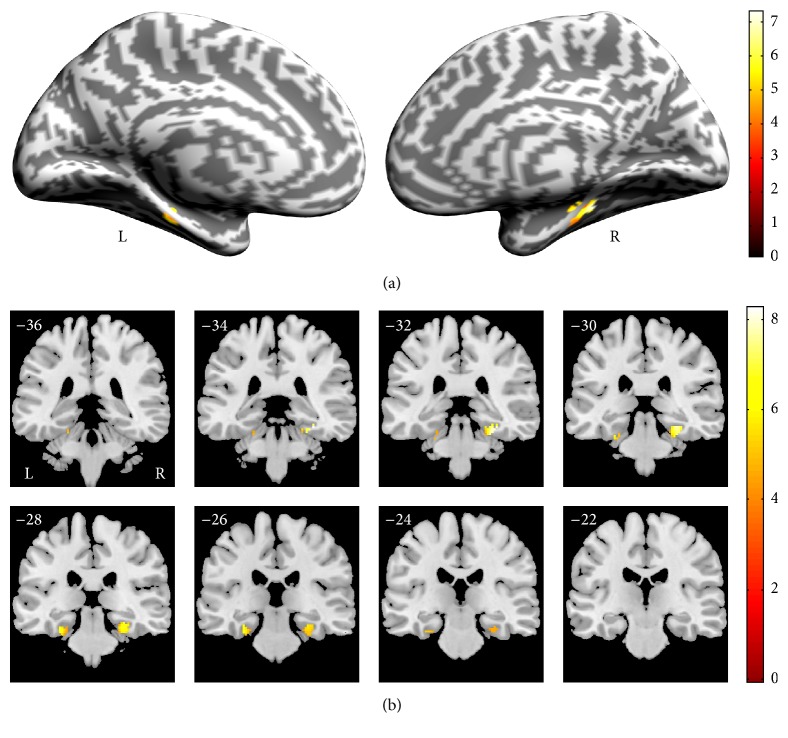
Random-effects statistical activation maps within the PHC for contextual shapes versus noncontextual shapes. (a) Cerebral cortex inflation of the left and right hemisphere. (b) Coronal sections. The numbers of coronal sections refer to the coordinates of coronal orientation in accordance with the Montreal Neurological Institute (MNI) space; L: left hemisphere; R: right hemisphere.

Figure 3(a) shows the results of the main-effect analysis [(CO + CS) versus (NO + NS)] where the contextual associative memory significantly activated the PHC regions compared with the noncontextual associative memory. The blood oxygenation level-dependent signal (BOLD) changes in the peak voxel in the left PHC and the right PHC are shown in Figures 3(b) and 3(c). After the main-effect analysis, we performed direct comparisons of CO versus NO (Figure 4) and CS versus NS (Figure 5), as shown in Table 2.

By comparing Figure 4 with Figure 5, we found that there were differences on the PHC activation between CO versus NO (left > right) and CS versus NS (right > left). To further analyze the results, we conducted the 3-way ANOVA on the BOLD signal changes of the left and right PHC. The main effects of three factors showed that the association was significant, *F*(1, 15) = 27.049, *P* < 0.0001; the semantics were not significant, *F*(1, 15) = 3.629, *P* = 0.076; and the hemisphere was not significant, *F*(1, 15) = 1.157, *P* = 0.299. The interaction between the two factors showed that the association × semantics was not significant, *F*(1, 15) = 0.000, *P* = 0.996; association × hemisphere was not significant, *F*(1, 15) = 1.479, *P* = 0.243; and semantics × hemisphere was significant, *F*(1, 15) = 64.526, *P* < 0.0001. The interaction of the three factors was also not significant, *F*(1, 15) = 1.872, *P* = 0.191. We also performed the comparisons in bilateral PHC. The results showed that, in the left PHC, CO versus NO [*F*(1, 15) = 14.937, *P* < 0.001] and CS versus NS [*F*(1, 15) = 9.634, *P* < 0.005] were significant; in the right PHC, CO versus NO [*F*(1, 15) = 11.918, *P* < 0.005] and CS versus NS [*F*(1, 15) = 17.832, *P* < 0.0001] were also significant. We further compared the left PHC and right PHC in four conditions: CO, CS, NO, and NS. The results showed, in CO, left PHC versus right PHC [*F*(1, 15) = 3.505, *P* < 0.05]; in CS, left PHC versus right PHC [*F*(1, 15) = 4.675, *P* < 0.05]; in NO, left PHC versus right PHC [*F*(1, 15) = 0.780, *P* = 0.385]; and, in NS, left PHC versus right PHC [*F*(1, 15) = 2.962, *P* = 0.096]. Otherwise, we also tested the involvement of PHC in [(CO + CS) versus (NO + NS)], CO versus CS, and NO versus NS, and no significant activation in PHC was found under all the three comparisons.

In addition, the comparison of these analyses, apart from the PHC, is shown in Table 3.

## 4. Discussion

To determine the role of the PHC in contextual associative information processing, we used two types of experimental materials to form the associative memory: real visual objects and meaningless visual shapes. In our study, we used the fMRI paradigm from Aminoff et al.'s study [31]. They created a novel learning paradigm to form new associations among meaningless visual patterns and investigated how the PHC mediates spatial and nonspatial associations by meaningless stimuli. Based on the fMRI paradigm, we selected visual objects with semantics and shapes without semantics to test the role of the PHC in associations processing and to address the different roles of bilateral PHC for the visual objects and visual shapes. The results showed that both CO and CS significantly activated the bilateral PHC more than the NO and NS. The left PHC was more activated than the right PHC under CO, whereas the right PHC was more activated than the left under CS. These results may be related to the different functions of the bilateral hemispheres. Previous studies suggested that the medial temporal lobe, including the hippocampus, is involved in semantic memory [41], and semantic memory is lateralized in the brain hemispheres [42, 43]. Using fMRI, Platel [44] investigated the brain activity of 14 subjects during semantic memory tasks. Semantic memory was found to be lateralized in the left brain. Some studies indicated that the left PHC exhibits greater activity for words than pseudowords [45–47]. In general, these studies support the idea that the left hemisphere is dominant for semantic memory. We found that the PHC is bilaterally more activated for CO than for NO and that the left PHC is more active than the right PHC, which suggests that semantic memory facilitates the formation of contextual associations of objects in the participants. In contrast, processing the associations from contextual objects, the associative memory with semantics significantly elicited the left PHC. This finding was verified by the results of our study. Meaningless CS also significantly activated the left PHC. No significant PHC activation for NO was observed compared with NS. We also found that the bilateral PHC was activated in CS compared with NS and that the right was greater than the left. In this study, the created objects were meaningless; thus, the formation of associations among the participants relied on the sensation of the visual images and not on semantic memory.

The involvement of the PHC in episodic memory has previously been suggested [17–23]. Two scenarios may be identified in our experiment: the learning scenario during the training session and the associative scenario from the stimuli. During the learning associations, two objects or shapes were presented together each time under contextual stimulus conditions. Two noncontextual objects or shapes were presented under noncontextual conditions, and these groups were presented only once. This phenomenon allowed for the same learning scenarios under both contextual and noncontextual conditions. Thus, the learning scenarios under the two conditions were disrupted when contextual conditions were compared with noncontextual conditions, which control episodic memory during the training phase. However, the two contextual objects or shapes also form a type of “associative scenario,” whereas the single noncontextual object or shape cannot form an “associative scenario.” In the fMRI experiment, when one contextual object or shape was presented, the cognitive process of the participants that determined whether the target was one of the associative groups was highly similar to the source memory in episodic memory rather than the single object or shape. Therefore, the contextual conditions activated the PHC more than noncontextual conditions.

This study also demonstrated that both contextual objects and shapes activated the posterior PHC. Numerous studies have demonstrated that the posterior PHC responds to spatial information [8–16]. Spatial information can be divided into the spatial relationship and spatial location. In the present study, both contextual objects and shapes significantly activated the posterior rather than the anterior PHC. This activation of the posterior PHC was unrelated to the spatial location, which is attributed to the random distribution of every two stimuli on the screen under all conditions, which did not bind the stimuli to a specific location. Therefore, the PHC activation cannot be attributed to the processing of the associations between the stimuli and their locations. Moreover, the posterior PHC responds to the associative processing of an “object” and its location [23, 34, 48]. Thus, when the spatial location is considered an “object,” the posterior PHC is attributed to associative processing between objects. Furthermore, both contextual objects and shapes significantly activated only the posterior PHC. This result may be attributed to the spatial processing required to form the contextual associations among objects or shapes.

In addition, other regions (including the hippocampus, lingual gyrus, precuneus, medial frontal lobe, and thalamus) were activated in both contextual objects and shapes (Table 3). Many previous studies have indicated that the hippocampus is more involved in associative learning and associative memory [1–7]. The lingual gyrus mainly involves the overall processing of the spatial information [49–51], and association processing is very important to the integrated processing of the information [33, 52]. The precuneus and medial frontal lobe are the core regions in the default network [53–55]. Bar et al. have found that there are the overlap between the network mediating contextual associations and the medial default network and proposed that the “default activity” and mind wandering rely on associative processing that is the unit of thought [56]. Otherwise, the thalamus is functionally connected to the hippocampus system [57] with respect to spatial memory and spatial sensory datum which are crucial for human episodic memory and rodent event memory [58, 59]. Taken together, these other activation instances are related to the associative processing.

In conclusion, PHC remains controversial in cognitive neuroscience. To gain insight into this issue, we used two types of experimental tasks to form associative memory: visual objects in reality and meaningless visual shapes. The results showed that both contextual objects and shapes significantly activated the bilateral PHC compared with noncontextual objects and shapes. This finding indicates that the PHC is more involved in contextual associations than in noncontextual associations. We also found that left PHC activation was higher than right PHC activation under the CO conditions, whereas activation in the right PHC was higher than the left under the CS conditions. In addition to the PHC, the hippocampal cortex, lingual gyrus, fusiform gyrus, precuneus, medial prefrontal cortex, and thalamus exhibited differential activity. In this study, the sample size is relatively small, which may affect the statistical validity. Thus, the sample size should be appropriately increased in further studies.

## Figures and Tables

**Table 1:** Accuracy of four conditions in the fMRI experiment (M ± SD).

Conditions	Accuracy (%)
Contextual objects (COs)	96.56 ± 3.64
Noncontextual objects (NOs)	95.63 ± 2.81
Contextual shapes (CSs)	94.06 ± 4.17
Noncontextual shapes (NSs)	94.69 ± 4.99

**Table 2:** Stereotactic coordinates and peak *z*-scores of the PHC activation during task comparisons.

Comparisons	Anatomical regions^a^	*x*	*y*	*z*	*z*-score	Cluster sizes (mm^3^)
CO versus NO	L PHC	−30	−32	−18	5.30	648
	L PHC	−32	−24	−24	5.12	
	R PHC	32	−30	−16	5.13	344
	R PHC	32	−24	−24	4.84	

CS versus NS	L PHC	−30	−28	−22	4.67	320
	L PHC	−22	−36	−20	3.94	
	R PHC	32	−34	−16	5.44	720

^a^The MNI coordinates of the centroid; L: left hemisphere; R: right hemisphere.

**Table 3:** Stereotactic coordinates and peak *z*-scores of the activation during task comparisons apart from the PHC.

Comparisons	Anatomical regions^a^	*x*	*y*	*z*	*z*-score	Cluster sizes (mm^3^)
CO versus NO	L hippocampus	−26	−6	−20	3.56	446
		−34	−10	−22	3.24	
	R hippocampus	36	−6	−24	3.99	213
		34	−14	−26	3.12	
	R lingual gyrus	26	−53	−6	5.13	140
		14	−74	−6	4.78	
		20	−64	−4	4.72	
	R precuneus	4	−46	62	3.85	89
		6	−46	70	3.81	
	L medial frontal lobe	−14	56	12	3.63	310
		−16	54	4	2.64	
	R medial frontal lobe	12	56	6	4.07	188
	R thalamus	20	−20	4	3.78	103

CS versus NS	L hippocampus	−34	−10	−22	3.87	141
		−32	−18	−22	3.54	
		−24	−8	−18	3.43	
	R hippocampus	36	−16	−22	4.59	462
		34	−14	−24	3.96	
		34	−4	−22	3.63	
	R lingual gyrus	16	−66	−4	5.41	219
		30	−48	−8	5.35	
		12	−42	−4	4.62	
	R precuneus	6	−46	58	4.19	117
		14	−40	46	3.13	
	L medial frontal lobe	−14	56	12	3.56	104
		−2	60	6	3.22	
	R medial frontal lobe	12	60	6	3.29	97
	L thalamus	−12	−32	0	3.27	53
		−20	−22	−2	2.85	
	R thalamus	10	−12	18	2.90	77

^a^The MNI coordinates of the centroid; L: left hemisphere; R: right hemisphere.
